# New Insights Into an Overlooked Entity: Long-Term Outcomes of Membranous Lupus Nephritis From a Single Institution Inception Cohort

**DOI:** 10.3389/fmed.2022.809533

**Published:** 2022-04-14

**Authors:** Eleni Kapsia, Smaragdi Marinaki, Ioannis Michelakis, George Liapis, Petros P. Sfikakis, Maria G. Tektonidou, John Boletis

**Affiliations:** ^1^Department of Nephrology and Renal Transplantation, Medical School, National and Kapodistrian University of Athens, Laiko Hospital, Athens, Greece; ^2^Department of Hygiene, Epidemiology and Medical Statistics, Medical School, National and Kapodistrian University of Athens, Athens, Greece; ^3^Department of Pathology, Medical School, National and Kapodistrian University of Athens, Laiko Hospital, Athens, Greece; ^4^Rheumatology Unit, First Department of Propaedeutic Internal Medicine, Medical School, National and Kapodistrian University of Athens, Laiko Hospital, Athens, Greece

**Keywords:** membranous, lupus nephritis, remission, flare, outcomes

## Abstract

**Introduction:**

Pure membranous lupus nephritis (MLN) accounts for 10–20% of total cases of lupus nephritis and is generally associated with a better patient and renal survival compared to proliferative classes. Studies of MLN are limited by small sample size and heterogeneity of included populations since patients with pure MLN and those with mixed classes are usually examined together.

**Aim of the Study:**

To describe clinical and laboratory characteristics of patients with pure MLN, therapeutic regimens, response to treatment, renal relapses, and their long-term renal survival and to define prognostic factors of remission and relapse.

**Methods:**

We retrospectively studied an inception cohort of 27 patients with histologically proven pure MLN. Clinical, laboratory and therapeutical parameters were recorded at diagnosis, at different time points (3–6–9–12–18–24–36–72 months) during the course of the disease, at time of renal flare, and at last follow up visit.

**Results:**

48.1% (13/27) of patients were treated with mycophenolic acid (MPA), 29.6% (8/27) with cyclophosphamide (CYC), and 3.7% (1/27) with cyclosporine (all in combination with corticosteroids). Five patients (18.5%) did not receive any immunosuppressive treatment. Mean duration of treatment was 4.7 ± 2.3 years. Median time to complete remission was 9 months (IQR = 7) and median time to partial remission was 4 months (IQR = 4). No clinical or laboratory parameter was found to be significantly associated with time to remission. Time to remission was not significantly affected by either of the two treatment regimens (CYC and MPA) (*p* = 0.43). Renal flare was observed in 6 (22%) of the 27 patients in a median time of 51 months (IQR = 63). Proteinuria >1 g/24 h at 1 year significantly correlated with risk of flare (OR 20, *p* = 0.02). After a median follow up period of 77 months, all patients had an eGFR > 60 ml/min/1.73 m^2^ (mean eGFR 100 ± 32 ml/min/1.73 m^2^).

**Conclusions:**

In a small cohort of patients with pure MLN, long-term renal survival was very good. With the limitation of the small sample size, we could not find any baseline clinical, biochemical or therapeutic factor that could predict time to remission. Proteinuria > 1 g/24 h at 1 year should be further examined in larger cohorts as a possible predictor of flare.

## Introduction

Renal involvement in systemic lupus erythematosus (SLE) may occur in 25–60% of patients during the course of the disease, and frequently at the time of first diagnosis of SLE (1–3). Lupus nephritis (LN) has long been considered as a major cause of morbidity and mortality in lupus patients (4).

Despite the knowledge gained in regard to the pathogenesis, clinical presentation and natural history of LN and the advances in treatment over the past decades, about 10–30% of LN patients will develop end stage renal disease (ESRD) (5, 6). Renal prognosis differs by race and ethnicity with African-Americans and Hispanics having worse renal outcomes than Asians and Caucasians (1–3). Lupus nephritis histological class is a major determinant of renal survival with up to 30% of patients with proliferative classes progressing to ESRD within 10 years compared to only 10% of those with membranous LN (7).

Pure membranous LN (MLN) accounts for 10–20% of total cases of LN and, although a better renal and patient prognosis compared to proliferative classes has been recognized, the risk of ESRD is not negligible ranging from 0 to 23% at 10 years in previous studies. Furthermore, MLN often presents with nephrotic syndrome which may be associated with thrombotic and infectious complications that negatively affect patients' morbidity and mortality (8–13).

Studies of membranous lupus nephritis (MLN) are often limited by the small samples, non- inception cohorts, the heterogeneity of examined populations and indirect evidence since composite data from pure MLN, and mixed MLN with proliferative classes are often presented. Also, there is limited evidence on prognostic factors of disease remission, relapse and long-term renal survival of patients with pure MLN and on optimal treatments.

The aim of the present study is: (a) to describe clinical and laboratory characteristics of patients with pure MLN, therapeutic approaches, response to treatment, and their long-term renal survival, and (b) to define prognostic factors of remission and flare.

## Patients and Methods

### Study Population

This is an inception cohort study of all patients with pure MLN diagnosed between 2001 and 2016 and followed at our joint academic center (Nephrology and Rheumatology Units) at Laiko Hospital until June 2019. All patients fulfilled the ACR classification criteria for SLE and lupus nephritis diagnosis was confirmed by renal biopsy. Pure MLN (class V) was classified according to the International Society of Nephrology/ Renal Pathology Society (ISN/RPS) 2003 lupus nephritis classification. Biopsies performed before 2003 were reassessed based on ISN/RPS 2003 classification system.

### Data Collection

Medical charts of patients were retrospectively reviewed and clinical, laboratory, and therapeutical parameters were recorded at the time of LN diagnosis and at 6–12–18–24–36–72 months after MLN diagnosis, at the time of renal flare (with or without a repeat biopsy) and at last follow-up visit. Patients with mixed MLN and <6 months of follow-up were excluded.

Data collected included demographic parameters, time from SLE diagnosis to LN onset, disease activity (expressed by Systemic Lupus Erythematosus Disease Activity Index 2000, SLEDAI-2K score) (14), anti-ds DNA titers, C3 and C4 levels, serum urea (Ur), creatinine (Cr) and albumin, eGFR (based on the CKD-EPI formula), 24-h proteinuria, urine sediment, renal biopsy histological parameters, and immunosuppressive treatment.

The study was approved by the Institutional Review Board (IRB) of Laiko General Hospital of Athens and Medical School of National Kapodistrian University of Athens. Due to the retrospective nature of the study, an informed consent was not required.

### Definitions

Remission and flare were defined according to the EULAR/ERA-EDTA (15) and the KDIGO recommendations (16). Active urine sediment was defined as the presence of >5 RBCs/hpf or ≥1 red cell casts. Complete remission (CR) was defined as proteinuria <500 mg/24 h and serum creatinine reduction within 10% from baseline. Partial remission (PR) was defined as ≥50% reduction in proteinuria to subnephrotic levels and serum creatinine within 10% from baseline. Nephritic flare was defined as an increase in glomerular haematuria by ≥10 RBCs/hpf with or without a decrease in GFR by ≥10%, irrespective of changes in proteinuria. Nephrotic flare was defined as reproducible doubling of proteinuria to >1,000 mg/24 h if complete response had been previously achieved or as reproducible doubling of proteinuria to ≥2,000 mg/24 h if partial response has been previously achieved. “Early” MLN was defined as onset of MLN <1 year from SLE diagnosis. “Late” MLN was defined as onset of MLN > 1 year from SLE diagnosis.

### Statistical Analysis

Continuous variables were expressed as the mean value and standard deviation or median value and interquartile range (IQR), whereas categorical variables as frequencies and percentages. To investigate the differences between baseline demographic, clinical, and laboratory variables between patients with different therapeutical schemes, the *t*-test and Mann–Whitney *U*-test for independent samples for continuous variables and the χ^2^ and Fisher exact test for categorical variables were applied. Univariate logistic regression analyses were performed to estimate the prognostic effect of various variables on the risk of renal flare and Cox regression analyses for investigating the association between the time to remission (either partial or complete) of patients with MLN and their clinical characteristics. Variables that were found to be significant (significance was set at α = 0.05) in the univariate analyses, as well as variables that showed to have a predictive role even though not strictly significant (*p* < 0.10), were included in the multivariate models. Since the number of flares recorded was small (only 6), multivariate logistic regression analysis could not be performed because it would be vulnerable to errors. The estimated odds ratios (ORs) and hazard ratios (HRs) of both the univariate and multivariate models, as well as the related *p*-values, are presented. Data were analyzed using Stata 13.0 software (Stata Corporation, College Station, TX). All tests proceeded as 2 tailed.

## Results

### Baseline Demographic, Clinical and Biochemical Parameters

The baseline demographic, clinical, laboratory, and histological parameters are shown in Table 1.

**Table 1:** Baseline demographic, clinical, and laboratory characteristics and treatment regimens.

**Baseline characteristics**	**Mean ±SD**,
	**median/IQR**,
	***N*/%**
Age (year) mean ± SD	47 ± 12
Sex (M–F) *N*/%	5/19–22/81
Duration of SLE (months) median/IQR	4/72
SLEDAI score mean ± SD	10.5 ± 4
Low C3 *N*/%	18/69.2
Low C4 *N*/%	16/61.5
Positive anti-dsDNA *N*/%	19/76
**Proteinuria (g/24 h) mean** **±SD**	4.9 ± 3.6
Proteinuria > 3 g/d *N*/%	18/67
Proteinuria 1–3 g/d *N*/%	6/22
Proteinuria <1 g/d *N*/%	3/11
Active urine sediment *N*/%	19/70
Hypertension *N*/%	4/14.8
Serum albumin (g/dl) mean ± SD	3.1 ± 0.8
**Serum Cr (mg/dl) mean** **±SD**	1 ± 1
eGFR (ml/min/1.73 m^2^)mean ± SD	111 ± 34
eGFR > 60 N/%	24/89
eGFR 30–60 *N*/%	1/3.7
eGFR <30 *N*/%	2/7.3
**Induction treatment**	
Mycophenolic acid *N*/%	13/48.1
Cyclophosphamide *N*/%	8/29.6
Cyclosporine *N*/%	1/3.7
None *N*/%	5/18.5
**Maintenance treatment**	
Mycophenolic acid *N*/%	18/66.7
Cyclophosphamide *N*/%	2/7.4
Cyclosporine *N*/%	1/3.7
Azathioprine *N*/%	1/3.7
None *N*/%	5/18.5
**Hydroxychloroquine**	
Yes *N*/%	8/29.6
No *N*/%	19/70.4
*Follow up (months) median/IQR*	*77/64*

*eGFR, estimated glomerular filtration rate using the CKD-EPI formula; SLEDAI, systemic lupus erythematosus disease activity index; anti-ds DNA, antibodies against double stranded DNA*.

Of note, all patients of the cohort were Caucasians.

### Treatment Regimens

All patients received ACE inhibitor or ARB. Thirteen patients (48.1%) were treated with mycophenolic acid (MPA), 8 (29.6%) with cyclophosphamide (CYC) and 1 (3.7%) with cyclosporine (all in combination with corticosteroids) (Table 1). Five patients (18.5%) did not receive any immunosuppressive treatment because of low grade proteinuria, according to the existing at that time recommendations. Eight (29.6%) patients were on hydroxychloroquine (HCQ) treatment at the time of LN diagnosis (Table 1). However, the majority of patients (22/27, 81.5%) received HCQ at some point during the course of their disease.

Patients treated with MPA and those treated with CYC differed significantly only in eGFR levels at baseline (Table 2). eGFR at baseline was lower in the CYC group (mean ± SD 74.6 ± 40.6) than in the MPA group (mean ± SD 110 ± 28.2) (*p* = 0.02).

**Table 2:** Comparison of baseline characteristics between the two treatment groups (mycophenolic acid vs. cyclophosphamide).

**Baseline characteristics**	**Treatment with mycophenolic acid (*N* = 13)**	**Treatment with cyclophosphamide (*N* = 8)**	***p*-value**
Age (year) mean ± SD	45 ± 15	50 ± 8	0.5
Sex (M–F) *N*/%	3/23–10/77	1/12.5–7/87.5	1
Duration of SLE (months) median/IQR	38/108	8/12	0.41
SLEDAI score mean ± SD	10.2/3.8	10.8/4.3	0.7
Low C3 *N*/%	8/67	6/75	1
Low C4 *N*/%	7/58	6/75	1
Positive anti-dsDNA *N*/%	8/66.7	6/75	1
**Proteinuria (g/24 h)** **mean** **±SD**	4.7 ± 2.7	6.5 ± 5.2	0.33
Proteinuria > 3 g/d *N*/%	9/69	6/75	1
Proteinuria 1–3 g/d *N*/%	3/23	1/12.5	
Proteinuria <1 g/d *N*/%	1/8	1/12.5	
Active urine sediment *N*/%	11/84.6	4/50	0.14
Hypertension *N*/%	1/7.6	2/25	0.53
Serum albumin (g/dl) mean ± SD	3.1 **±** 0.8	2.8 ± 0.7	0.49
Serum Cr (mg/dl) mean ± SD	0.7 ± 0.24	1.7 ± 1.6	0.056
**eGFR (ml/min/1.73 m**^**2**^**) mean** **±SD**	110 ± 28.2	74.6 ± 40.6	**0.02**
eGFR > 60 *N*/%	12/92	6/75	0.13
eGFR 30–60 *N*/%	1/8	–	
eGFR <30 *N*/%	–	2/25	
Duration of treatment (months) mean ± SD	4.4 ± 2.8	5.2 ± 3	0.56

*eGFR, estimated glomerular filtration rate using the CKD-EPI formula; SLEDAI, systemic lupus erythematosus disease activity index; anti-ds DNA, antibodies against double stranded DNA*.

*Bold values are those with statistical significance (p <0.05)*.

Mean duration of treatment was 4.7 ± 2.3 years and did not differ significantly between the two treatment groups.

When we divided patients into those with “early” (<1 year since SLE diagnosis) onset of MLN and those with “late” (>1 year since SLE diagnosis) onset of MLN, we found that the two groups differed significantly in regard to baseline proteinuria. “Early” MLN patients had a median baseline proteinuria of 5.5 g/d (IQR = 4.3) vs. 2.7 g/d (IQR = 2.1) in “late” MLN (*p* = 0.03) (Table 3).

**Table 3:** Comparison of baseline characteristics between “early” (<1 year) and “late” (>1 year) LMN patients.

**Baseline characteristics**	**Early MLN (*N* = 14)**	**Late MLN (*N* = 13)**	***p*-value**
Age (year) mean ± SD	44 ± 11	50 ± 11	0.11
Sex (M–F) N/%	2/14–12/86	3/23–10/77	0.6
Duration of SLE (months) median/IQR	0/1	72/84	<0.01
SLEDAI score median/IQR	11.7/3.9	9.3/3.6	0.08
Low C3 *N*/%	11/78	7/58	0.4
Low C4 *N*/%	9/64	7/58	1
Positive dsDNA *N*/%	11/78	8/72	1
**Proteinuria (g/24 h) median/IQR**	5.5/4.3	2.7/2.1	**0.03**
Proteinuria > 3 g/d *N*/%	12/86	6/47	0.08
Proteinuria 1–3 g/d *N*/%	1/7	5/38	
Proteinuria <1 g/d *N*/%	1/7	2/15	
Active urine sediment *N*/%	10/72	9/69	1
Hypertension *N*/%	3/21	1/7	0.6
Serum albumin (g/dl) mean ± SD	2.9 ± 0.8	3.4 ± 0.6	0.23
Serum Cr (mg/dl) mean ± SD	0.6 ± 0.6	0.7 ± 0.2	0.63
**eGFR (ml/min/1.73 m**^**2**^**) mean** **±SD**	96.1 ± 41.3	103 ± 25	0.7
eGFR > 60 *N*/%	12/86	12/92	0.4
eGFR 30–60 *N*/%	–	1/8	
eGFR <30 *N*/%	2/14	–	
Duration of treatment (years) mean ± SD	5 ± 2.7	3.7 ± 1.9	0.31
**Induction treatment**			
Mycophenolic acid *N*/%	7/50	6/46	1
Cyclophosphamide *N*/%	4/28	4/30	1
Cyclosoprine N/%	–	2/15	0.2

*eGFR, estimated glomerular filtration rate using the CKD-EPI formula; SLEDAI, systemic lupus erythematosus disease activity index; anti-ds DNA, antibodies against double stranded DNA*.

*Bold values are those with statistical significance (p <0.05)*.

### Remission Rates and Prognostic Factors of Remission

**Figure 1:**
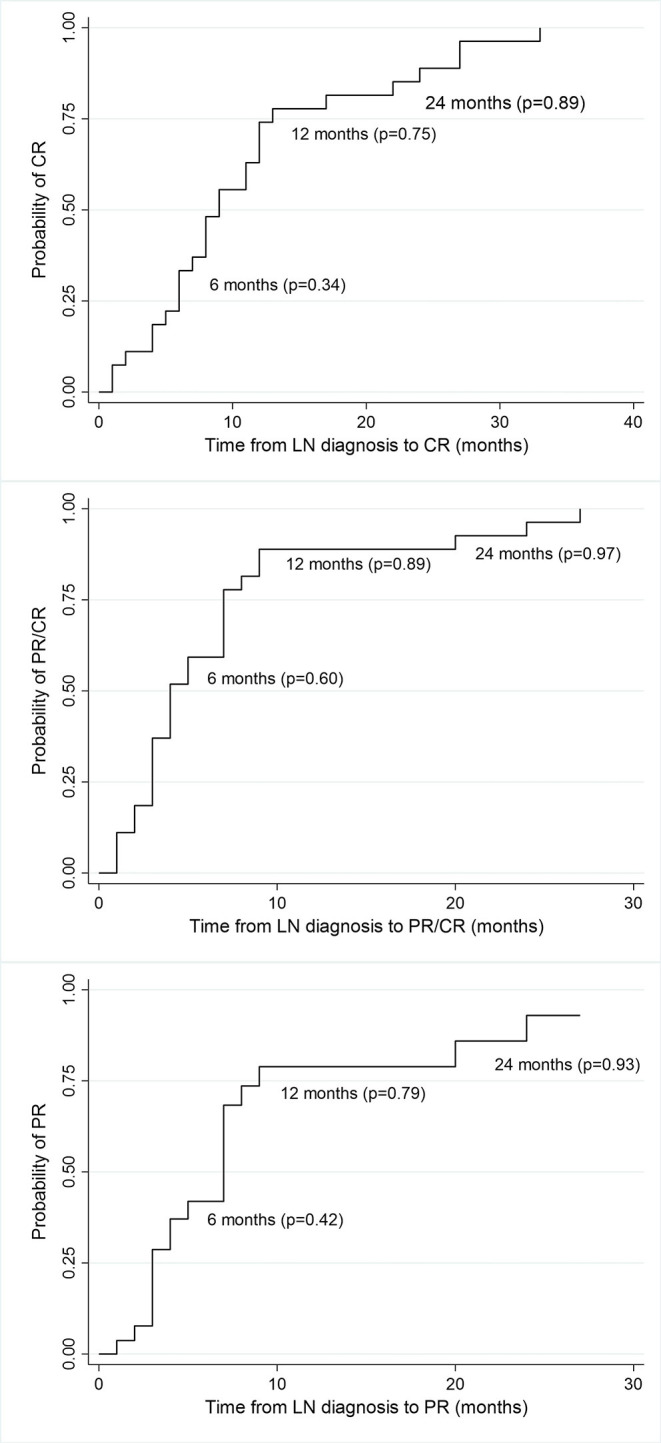
Kaplan-Meier estimates of survival probability of partial and complete remission at different time points from LN diagnosis. CR, complete remission; PR, partial remission; p, probability.

At 6 months, 77% of the total cohort achieved remission (37% CR, 40% PR). At 12 months, 89% were in remission (70% CR, 19% PR) and at the end of follow-up (median 77 months), all patients were in remission (89% CR, 11%PR) (Figure 1).

**Figure 2:**
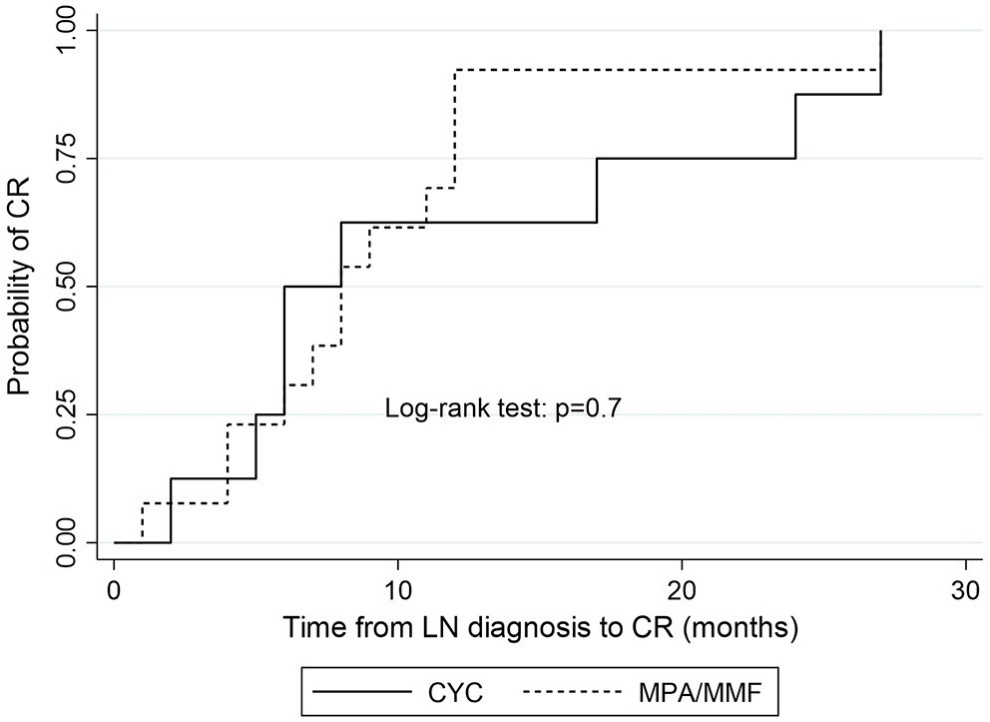
Kaplan-Meier estimates of survival probability of complete remission according to induction treatment. CYC, cyclophosphamide; MPA, mycophenolic acid; CR, complete remission.

The median time to complete remission was 9 months (IQR = 7) and the median time to partial remission was 4 months (IQR = 4). Median time to remission did not differ significantly between patients treated with MPA acid and those treated with CYC. Median time to complete remission in MPA group was 8 months (IQR = 6) vs. 6 months (IQR = 18) in CYC group (*p* = 0.84; Figure 2), median time to partial remission was 3 months (IQR = 2) in both treatment arms (*p* = 0.48), and median time to either complete or partial remission was 3 months (IQR = 2) in both groups (*p* = 0.48).

**Figure 3:**
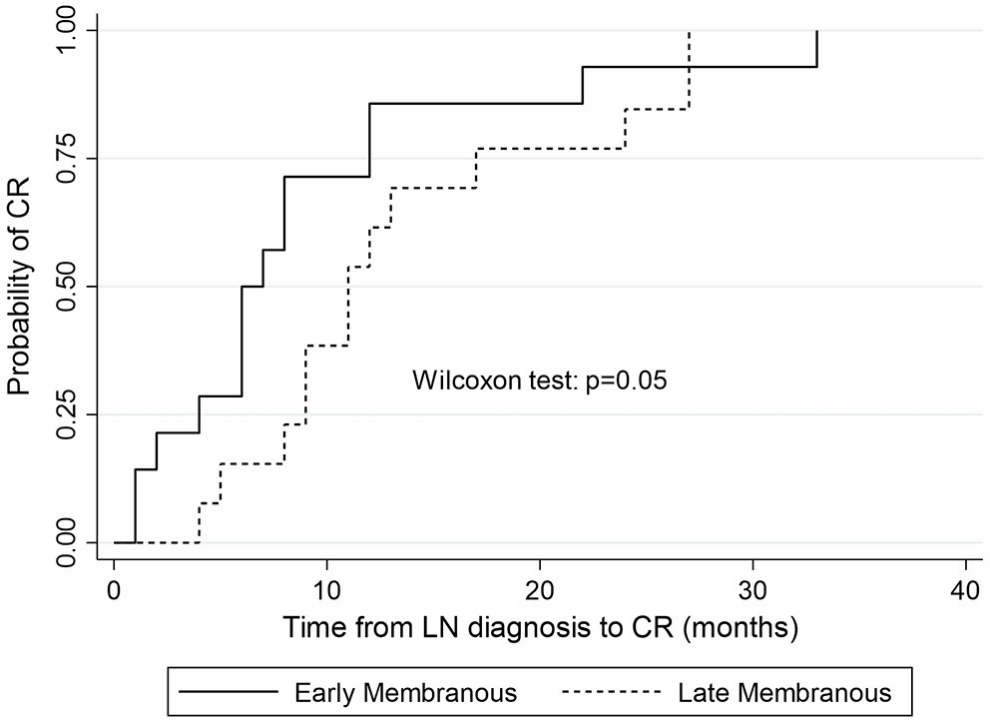
Kaplan-Meier estimates of survival probability of complete remission in “early” vs. “late” MLN.

Time to complete remission differed significantly between patients with “early” MLN (median 6.5 months, IQR = 8) and those with “late” MLN (median 11 months, IQR = 8; *p* = 0.05; Figure 3).

In Cox regression analysis no clinical or laboratory parameter was found to be significantly associated with time to remission (CR or PR; Table 4). Neither of the two treatment regimens (CYC and MPA) correlated to time to remission (HR = 0.69, *p* = 0.43). No significant correlation was found between onset of MLN (“early” vs. “late”) and remission time (HR = 0.61, *p* = 0.22).

**Table 4:** Correlations of clinical, laboratory, and treatment parameters with time to remission (either partial or complete).

**Variables**	**Univariate models**		**Multivariate model**	
	**HR**	**95% CIs (*p*-value)**	**HR**	**95% CIs (*p*-value)**
**eGFR at diagnosis (ml/min/1.73 m** ^**2**^ **)**				
<30	Reference Group			
31–60	0.18	0.01, 2.23 (0.18)	0.37	0.02, 5.25 (0.46)
>60	0.22	0.04, 1.06 (0.06)	0.26	0.05, 1.29 (0.1)
**Proteinuria at diagnosis (g/day)**				
<1	Reference Group			
1–3	1.95	0.37, 10.2 (0.42)		
>3	2.01	0.45, 8.9 (0.35)		
Age (years)	1.01	0.97, 1.04 (0.49)		
**Sex**				
Male	Reference Group			
Female	0.58	0.21, 1.6 (0.29)		
Diagnosis of SLE to LN (years)	0.98	0.89, 1.07 (0.68)		
**Time of LN after SLE diagnosis**				
Early (<1 year)	Reference Group			
Late (>1 year)	0.61	0.28, 1.34 (0.22)		
SLEDAI score	1.1	0.98, 1.24 (0.099)		
**Induction treatment**				
Cyclophosphamide	Reference Group			
Mycophenolic acid	0.69	0.28, 1.71 (0.43)		
**Hypertension**				
No	Reference Group			
Yes	2.26	0.75, 6.79 (0.144)		
**Low C3**				
No	Reference Group			
Yes	2.37	0.98, 5.7 (0.054)	2.28	0.45, 11.5 (0.31)
**Low C4**				
No	Reference Group			
Yes	2.13	0.93, 4.9(0.07)	1.02	0.22, 4.65 (0.97)
**Anti-ds-DNA**				
Negative	Reference Group			
Positive	0.69	0.93, 4.9 (0.45)		

*eGFR, estimated glomerular filtration rate using the CKD-EPI formula; SLEDAI, systemic lupus erythematosus disease activity index; anti-ds DNA, antibodies against double stranded DNA*.

### Renal Flares and Prognostic Factors of Flare

**Figure 4:**
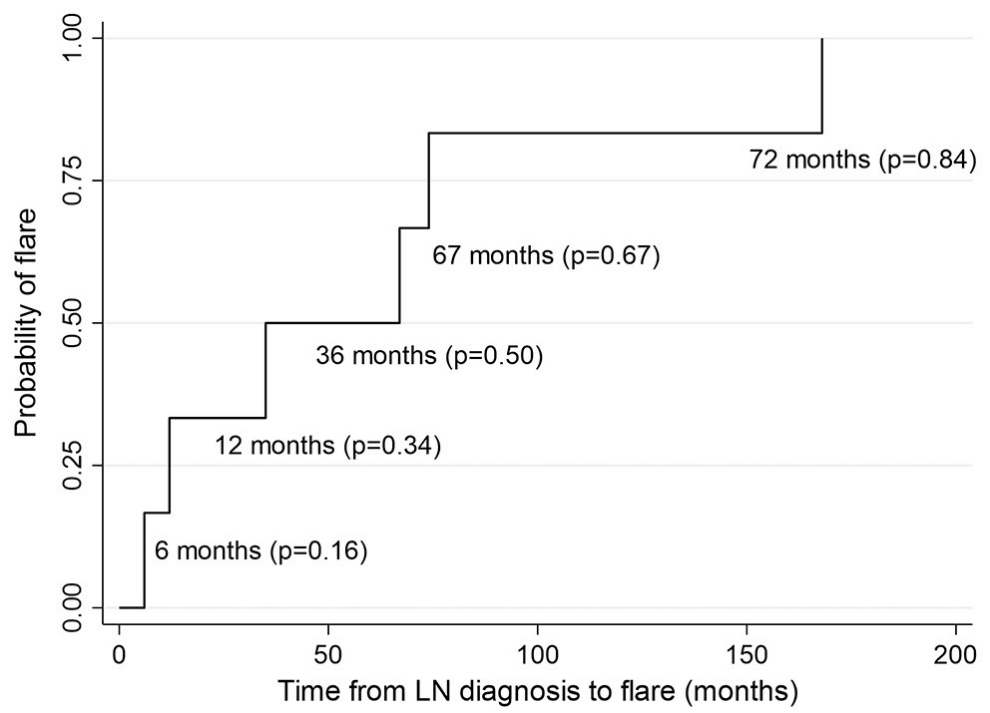
Kaplan-Meier estimates of survival probability of renal flare at different time points from LN diagnosis. P, probability.

Renal flare was observed in 6 (22%) of the 27 patients in a median time of 51 months (IQR = 63; Figure 4).

Only one of these flares was nephritic and five were nephrotic. Among the patients who had a flare, 2 (33%) had been treated with CYC, 2 (33%) with MPA, 1 (17%) with cyclosporine while 1 patient (17%) had never received any immunosuppressive treatment. In 3 (50%) of six cases no repeat biopsy was performed. In the other three cases, the repeat biopsy did not reveal a class switch.

In univariate logistic regression analysis, proteinuria>1 g/24 h at 1 year significantly correlated with risk of flare (OR = 20, *p* = 0.02; Table 5). eGFR > 60 ml/min/1.73 m^2^ at diagnosis, proteinuria 1–3 g/24 h at diagnosis, female sex and treatment with MPA were associated with a lower risk of flare but not in a statistically significant manner. Hypertension and low C3 and C4 levels at diagnosis were associated with increased risk of flare but this correlation wasn't statistically significant. Multivariate analysis was not possible due to small number of events.

**Table 5:** Prognostic factors of renal flare.

**Variables**	**Univariate models**			
	**OR**	**95% CIs (*p*-value)**		
**eGFR at diagnosis (ml/min/1.73 m** ^**2**^ **)**				
<30	Reference Group			
31–60	–	–		
>60	0.26	0.01, 4.98 (0.37)		
Time to PR (months)	1	0.87, 1.15 (0.94)		
Time to CR (months)	1.05	0.94, 1.16 (0.34)		
Time to PR/CR (months)	1	0.87, 1.13 (0.94)		
**Proteinuria at diagnosis (g/day)**				
**<1**	Reference Group			
**1–3**	0.52	0.04, 5.62 (0.59)		
**>3**	–	–		
**Proteinuria at 12 months (g/day)**				
<1	Reference Group			
>1	**20**	**1.53, 260 (0.02)**		
Age (years)	1	0.93, 1.08 (0.85)		
**Sex**				
Male	Reference Group			
Female	0.33	0.04, 2.69 (0.30)		
Diagnosis of SLE to LN (years)	0.98	0.96, 1.01 (0.31)		
**Time of LN after SLE diagnosis**				
Early (<1 year)	Reference Group			
Late (>1 year)	0.45	0.06, 3.04 (0.41)		
SLEDAI score	1.07	0.83, 1.37 (0.57)		
**Induction treatment**				
Cyclophosphamide	Reference Group			
Mycophenolic acid	0.54	0.06, 4.91 (0.58)		
**Hypertension**				
No	Reference Group			
Yes	1.2	0.10, 14.1 (0.88)		
**Low C3**				
No	Reference Group			
Yes	2.69	0.26, 27.8 (0.40)		
**Low C4**				
No	Reference Group			
Yes	4.09	0.40, 41.6 (0.23)		
**Anti-dsDNA**				
Negative	Reference Group			
Positive	–	–	–	–

*eGFR, estimated glomerular filtration rate using the CKD-EPI formula; SLEDAI, systemic lupus erythematosus disease activity index; anti-ds DNA, antibodies against double stranded DNA; CR, complete remission; PR, partial remission*.

*Bold values are those with statistical significance (p <0.05)*.

### Renal and Patient Survival

No patient in our cohort developed ESRD in a median follow up period of 77 months. In fact, all patients at the end of follow up had an eGFR>60 ml/min/1.73 m^2^ (mean eGFR 100 ± 32 ml/min/1.73 m^2^). Notably, 89% of the patients had an eGFR>60 ml/min/1.73 m^2^ at the time of MLN diagnosis.

At the end of follow up, all patients were in remission (89% CR, 11% PR), and mean 24 h proteinuria was 0.12 ± 0.12 g. Ten (37%) of 27 patients did not receive any immunosuppressive drug, 13 (48%) continued immunosuppressive treatment (10 on MPA, 3 on AZA) and 4 (15%) were on corticosteroids only.

At a median follow up time of 77 months, no death was recorded but there were 10 patients lost to follow up after 3 years (all in remission).

### Adverse Events

No thrombotic or cardiovascular event occurred. One episode of herpes zoster and one episode of HBV reactivation were successfully managed with antiviral therapy and temporary reduction of immunosuppression.

## Discussion

Knowledge gained in the field of pure MLN shows a favorable renal prognosis (compared to proliferative and mixed forms of LN) and underscores the need of treatment regimens consisting of a combination of corticosteroids and an immunosuppressive agent, even in patients with subnephrotic levels of proteinuria. The optimal immunosuppressant agent as well as the optimal duration of treatment are not yet fully elucidated. Achievement of remission, time till remission and flare occurrence have been recognized to affect the long-term renal outcome in LN patients. However, the issue of factors that could predict time to remission and flare occurrence in the subpopulation of patients with pure MLN has not been fully addressed. Time to remission (and not only achievement of remission *per se*) is of great importance in these patients, since longer time to remission exposes them to a greater risk of thrombotic and infectious complications associated with the high levels of proteinuria patients with pure MLN have. We have showed that no baseline clinical or laboratory parameter (not even the level of baseline proteinuria) could serve as a prognostic factor of time to remission and that both major treatment regimens (MPA and CYC) induced remission in similar times. Twelve-month proteinuria has been recognized as a predictor of long-term renal survival in the total cohort of LN patients. Our study suggests that 12-month proteinuria can be also used as a prognostic factor of flare in pure MLN patients. Renal survival of patients with MLN ranges from 96 to 98% at 5 years, 72–100% at 10 years, and reaches 83% at 15 years (7, 17–22). Nevertheless, in certain ethnic groups, such as African Americans, it may be significantly lower (71% at 5 years) (23). In accordance with previous studies, renal survival in our cohort was excellent with all of the patients having an eGFR > 60 ml/min/1.73 m^2^ at a median follow up time of 77 months. Even the three patients who had an eGFR <60 ml/min/1.73 m^2^ at diagnosis (2 of them with eGFR <20 ml/min/1.73 m^2^) managed to improve their renal function during the first 12 months. Renal injury in these patients can be attributed to hemodynamic changes caused by proteinuria and as the latter resolved with treatment, renal function recovered. The excellent renal survival in our cohort can be attributed to the fact that all patients were Caucasians and the majority had normal renal function at diagnosis. The fact that these patients were very closely followed up at a center highly experienced in the management of LN may have also contributed to the good renal outcomes (24), as it was previously shown (17).

The optimal treatment for MLN has not yet been fully elucidated. There is, however, strong evidence that a combination of corticosteroids and immunosuppressives is superior to steroid monotherapy (25, 26). Azathioprine has been shown to be effective in achieving remission but it was associated with high relapse rates (18, 19). Several studies have reported similar rates of clinical response between MPA and CYC (27–29) while others, including a network meta-analysis, have showed superiority of MMF over CYC (21, 30, 31). Calcineurin inhibitors have also been used to treat pure MLN with similar overall response compared to MMF, CYC or AZA (26, 32–35). CNIs are often associated with a faster resolution of proteinuria (35) but also with a higher relapse rate compared to MMF or CYC (32). These observations may, in part, be explained by the fact that CNIs, besides their immunosuppressive actions, affect the intraglomerular pressure and act on the podocytes' cytoskeleton leading to proteinuria reduction (36). In our cohort, 48.1% of patients were treated with MPA, 29.6% with CYC, and only one patient with cyclosporine. The two treatment groups (MPA and CYC) differed significantly in baseline eGFR levels, with patients in the CYC group having worse renal function at baseline compared to MPA group. In regression analysis and after adjustment for baselineeGFR, neither treatment correlated with time to remission or risk of flare, implying that, in white patients with relatively preserved renal function, both treatments are equally effective.

Several studies have demonstrated that achievement of remission, time till remission and development of flares are factors significantly associated with worse long-term renal outcome (17, 37–41). All patients included in our study achieved remission at some point during the disease course. Interestingly, baseline proteinuria, which has been suggested to predict remission (25), did not seem to affect time to remission in our cohort and neither did the therapeutic regimen applied. Renal function at baseline did not also prove to be a significant predictor of time to remission but it should be noted that 89% (24/27) of the patients had normal renal function (eGFR > 60 ml/min/1.73 m^2^) at the time of MLN diagnosis. No other baseline clinical or biochemical parameter has emerged as a significant prognostic factor of time to remission, possibly due to the small number of patients studied.

Renal flares in pure MLN patients range from 22 to 45% in different studies (17, 19, 21, 22) while in our cohort they occurred in only 22% of patients. Proteinuria >1 g/24 h at 1 year after the diagnosis appeared to be the only statistically significant risk factor for flare. This observation adds to the value of 12-month proteinuria, which has, in recent years, emerged as a more reliable predictor of long-term renal outcomes in LN patients (38, 39, 41, 42). Therapeutic regimen (MPA or CYC) did not seem to affect the risk of flare, neither did the time to remission. Lower C3 and C4 levels appeared to increase the risk of flare but not significantly. Larger studies are needed to further examine C3 and C4 as potential determinants of flare occurrence in pure MLN.

There is lack of data in the literature in regard to how the time of onset of MLN affects its clinical presentation as well as its response to treatment and long-term renal outcome. We decided to divide our patients into those presenting with MLN at the time of SLE diagnosis or at some time during the following 12 months (“early” MLN) and into those presenting with MLN afterwards (“late” MLN). Although patients with “early” disease had higher levels of proteinuria at baseline than patients with “late” disease, the former achieved complete remission sooner than the latter, a finding that deserves further investigation.

Our cohort reflects a real-world, uniform management of pure MLN patients followed at a dedicated, specialized center with available data for all patients for a median follow-up period of 77 months. Another strength of the study is its inception cohort design which contributes, par excellence, to the definition of the natural history of a disease and to the determination of correlations between a certain outcome and prognostic factors. Our study has several limitations such as its retrospective nature and mainly, the small number of patients. Such a small sample size is not able to ensure the statistical power of the results, which should be interpreted with caution and evaluated in larger cohorts. Since MLN is a rare entity and progresses rather slowly, multicentric cohorts with longer follow up are needed.

## Conclusions

Pure MLN in our cohort was associated with very good long-term renal outcomes. Mycophenolic acid and cyclophosphamide seemed to be equally effective in means of time to remission and flare prevention. With the limitation of the small sample size, we could not find any baseline clinical or biochemical factor that could predict time to remission. Proteinuria >1 g/24 h at 1 year seemed to be associated with a higher risk of renal flare but this observation should be further examined in larger cohorts.

## Data Availability Statement

The raw data supporting the conclusions of this article will be made available by the authors, without undue reservation.

## Ethics Statement

The studies involving human participants were reviewed and approved by Institutional Review Board (IRB) of Laiko General Hospital of Athens and Medical School of National Kapodistrian University of Athens. Written informed consent for participation was not required for this study in accordance with the national legislation and the institutional requirements.

## Author Contributions

MT, JB, and PS designed the study. EK collected the data and drafted the article. GL reviewed the histological data. IM analyzed the data. SM and MT supervised the process. MT, SM, PS, and JB reviewed the final version of the article. All authors provided contributions and approved the version of the article to be published.

## Conflict of Interest

The authors declare that the research was conducted in the absence of any commercial or financial relationships that could be construed as a potential conflict of interest.

## Publisher's Note

All claims expressed in this article are solely those of the authors and do not necessarily represent those of their affiliated organizations, or those of the publisher, the editors and the reviewers. Any product that may be evaluated in this article, or claim that may be made by its manufacturer, is not guaranteed or endorsed by the publisher.

## References

[1] Seligman VA Lum RF Olson JL Li H Criswell LA. Demographic differences in the development of lupus nephritis: a retrospective analysis. Am J Med. (2002) 112:726–9. 10.1016/S0002-9343(02)01118-X 12079714

[2] Bastian HM Roseman JM McGwin G Jr Alarcón GS Friedman AW Fessler BJ. Lupus in minority populations: nature vs nurture. Systemic lupus erythematosus in three ethnic groups. XII. Risk factors for lupus nephritis after diagnosis. Lupus. (2002) 11:152–60. 10.1191/0961203302lu158oa 12004788

[3] Korbet SM Schwartz MM Evans J Lewis EJ Collaborative Study Group. Severe lupus nephritis: racial differences in presentation and outcome. J Am Soc Nephrol. (2007) 18:244–54. 10.1681/ASN.2006090992 17167111

[4] Doria A Iaccarino L Ghirardello A Zampieri S Arienti S Sarzi-Puttini P. Long-term prognosis and causes of death in systemic lupus erythematosus. Am J Med. (2006) 119:700–6. 10.1016/j.amjmed.2005.11.034 16887417

[5] Lateef A Petri M. Unmet medical needs in systemic lupus erythematosus. Arthritis Res Ther. (2012) 14(Suppl. 4):S4. 10.1186/ar3919 23281889 PMC3535719

[6] Croca SC Rodrigues T Isenberg D. Assessment of a lupus nephritis cohort over a 30-year period. Rheumatology (Oxford). (2011) 50:1424–30. 10.1093/rheumatology/ker101 21415024

[7] Tektonidou M Dasgupta A Ward MM. Risk of end-stage renal disease in patients with lupus nephritis, 1971-2015: a systematic review and Bayesian meta-analysis. Arthritis Rheumatol. (2016) 68:1432–41. 10.1002/art.39594 26815601 PMC5071782

[8] Austin HA Illei GG. Membranous lupus nephritis. Lupus. (2005) 14:65–71. 10.1191/0961203305lu2062oa 15732291

[9] Mok CC. Membranous nephropathy in systemic lupus erythematosus: a therapeutic enigma. Nat Rev Nephrol. (2009) 5:212–20. 10.1038/nrneph.2009.14 19322186

[10] Daleboudt GM Bajema IM Goemaere NN van Laar JM Bruijin JA Berger SP. The clinical relevance of a repeat biopsy in lupus nephritis flares. Nephrol Dial Transplant. (2009) 24:3712–17. 10.1093/ndt/gfp359 19622571

[11] Pagni F Galimberti S Goffredo P Basciu M Malachina S Pilla D. The value of repeat biopsy in the management of lupus nephritis: an international multicentre study in a large cohort of patients. Nephrol Dial Transplant. (2013) 28:3014–23. 10.1093/ndt/gft272 23975838

[12] Miyake K Akahoshi M Nakashima H. Th subset balance in lupus nephritis. J Biomed Biotechnol. (2011) 2011:980286. 10.1155/2011/980286 21904445 PMC3163408

[13] Dalton K Smith M Thurman JM. The development of membranous lupus nephritis during treatment with mycophenolate mofetil for proliferative renal disease. NDT Plus. (2010) 3:346–8. 10.1093/ndtplus/sfq046 25949427 PMC4421507

[14] Gladman DD Ibanez D Urowitz MB. Systemic lupus erythematosus disease activity index 2000. J Rheumatol. (2002) 29:288–91. 11838846

[15] Bertsias GK Tektonidou M Amoura Z Aringer M Bajema I Berden JHM. Joint European League Against Rheumatism and European Renal Association-European Dialysis and Transplant Association (EULAR/ERA-EDTA) recommendations for the management of adult and paediatric lupus nephritis. Ann Rheum Dis. (2012) 71:1771–82. 10.1136/annrheumdis-2012-201940 22851469 PMC3465859

[16] Kidney Disease: Improving Global Outcomes (KDIGO) Glomerulonephritis Work Group. KDIGO clinical practice guideline for glomerulonephritis. Kidney Int Suppl. (2012) 2:139–274. 10.1038/kisup.2012.9

[17] Moroni G Quaglini S Gravellone L Gallelli B Leoni A Messa P. Membranous nephropathy in systemic lupus erythematosus: long-term outcome and prognostic factors of 103 patients. Semin Arthritis Rheum. (2012) 41:642–51. 10.1016/j.semarthrit.2011.08.002 22285127

[18] Mok CC Ying KY Lau CS Yim CW Ng WL Wong WS. Treatment of pure membranous lupus nephropathy with prednisone and azathioprine: an open-label trial. Am J Kidn Dis. (2004) 43:269–76. 10.1053/j.ajkd.2003.10.029 14750092

[19] Mok CC Ying KY Yim CW Ng WL Wong WS. Very long-term outcome of pure lupus membranous nephropathy treated with glucocorticoid and azathioprine. Lupus. (2009) 18:1091–5. 10.1177/0961203309106602 19762384

[20] Farinha F Pepper RJ Oliveira DG McDonell T Isenberg DA Rahman A. Outcomes of membranous and proliferative lupus nephritis-analysis of a single-centre cohort with more than 30 years of follow-up. Rheumatology (Oxford). (2020) 59:3314–23. 10.1093/rheumatology/keaa103 32303057 PMC7590413

[21] Mejía-Vilet JM Córdova-Sanchez BM Uribe-Uribe NO Correa-Rotter R. Immunosuppressive treatment for pure membranous lupus nephropathy in a Hispanic population. Clin Rheumatol. (2016) 35:2219–27. 10.1007/s10067-016-3366-y 27475791

[22] Sun HO Hu WX Xie HL Zhang HT Chen HP Zeng CH. Long-term outcome of Chinese patients with membranous lupus nephropathy. Lupus. (2008) 17:56–61. 10.1177/0961203307083443 18089685

[23] Okpechi IG Ayodele OE Duffield M Swanepoel CR. Outcome of patients with membranous lupus nephritis in Cape Town South Africa. Nephrol Dial Transplant. (2012) 27:3509–15. 10.1093/ndt/gfs122 22610989

[24] Ward MM. Association between physician volume and in-hospital mortality in patients with systemic lupus erythematosus. Arthritis Rheum. (2005) 52:1646–54. 10.1002/art.21053 15934091

[25] Austin HA Illei GG Braun MJ Ballow JE. Randomized, controlled trial of prednisone, cyclophosphamide and cyclosporine in lupus membranous nephropathy. J Am Soc Nephrol. (2009) 20:901–11. 10.1681/ASN.2008060665 19297556 PMC2663831

[26] Swan JT Riche DM Riche KD Majithia V. Systematic review and meta-analysis of immunosuppressant therapy clinical trials in membranous lupus nephritis. J Invest Med. (2011) 59:246–58. 10.2310/JIM.0b013e318204c965 21328792

[27] Radhakrishnan J Moutzouris DA Ginzler EM Solomons N Siempos II Appel GB. Mycophenolate mofetil and intravenous cyclophosphamide are similar as induction therapy for class V lupus nephritis. Kidney Int. (2010) 77:152–60. 10.1038/ki.2009.412 19890271

[28] Appel GB Contreras G Dooley MA Ginzler EM Isenberg D Wofsy D. Mycophenolate mofetil versus cyclophosphamide for induction treatment of lupus nephritis. J Am Soc Nephrol. (2009) 20:1103–12. 10.1681/ASN.2008101028 19369404 PMC2678035

[29] Rathi M Goyal A Jaryal A Sharma A Gupta PK Ramachandran R. Comparison of low-dose intravenous cyclophosphamide with oral mycophenolate mofetil in the treatment of lupus nephritis. Kidney Int. (2016) 89:235–42. 10.1038/ki.2015.318 26489028

[30] Rivera TL Belmont HM Malani S Latorre M Benton L Weisstuch J. Current therapies for lupus nephritis in an ethnically heterogenous cohort. J Rheumatol. (2009) 36:298–305. 10.3899/jrheum.080335 19040310

[31] Tang KT Tseng CH Hsieh TY Chen DY. Induction therapy for membranous lupus nephritis: a systematic review and network meta-analysis. Int J Rheum Dis. (2018) 21:1163–72. 10.1111/1756-185X.13321 29879319

[32] Mok CC Ying KY Yim CW Siu YP Tong KH To CH. Tacrolimus versus mycophenolate mofetil for induction therapy of lupus nephritis: a randomized controlled trial and long-term follow-up. Ann Rheum Dis. (2016) 75:30–6. 10.1136/annrheumdis-2014-206456 25550339

[33] Yap DY Yu X Chen XM Lu F. Chen N Li XW. Pilot 24 month study to compare mycophenolate mofetil and tacrolimus in the treatment of membranous lupus nephritis with nephrotic syndrome. Nephrology (Carlton). (2012) 17:352–57. 10.1111/j.1440-1797.2012.01574.x 22295934

[34] Zhang X Ji L Yang L Tang X Qin W. The effect of calcineurin inhibitors in the induction and maintenance treatment of lupus nephritis: a systematic review and meta-analysis. Int Urol Nephrol. (2016) 48:731–43. 10.1007/s11255-015-1201-z 26781720

[35] Szeto CC Kwan BCH Lai FMM Tam LS Li EKM Chow KM. Tacrolimus for the treatment of systemic lupus erythematosus with pure class V nephritis. Rheumatology. (2008) 47:1678–81. 10.1093/rheumatology/ken335 18753192

[36] Rafael-Vidal C Altabás I Pérez N Rodríguez CM Pego-Reigosa JM Garcia S. Calcineurin and systemic lupus erythematosus: the rationale for using calcineurin inhibitors in the treatment of lupus nephritis. Int J Mol Sci. (2021) 22:1263. 10.3390/ijms22031263 33514066 PMC7865978

[37] El Hachmi M Jadoul M Lefèbvre C Depresseux G Houssiau FA. Relapses of lupus nephritis: incidence, risk factors, serology and impact on outcome. Lupus. (2003) 12:692–6. 10.1191/0961203303lu444oa 14514132

[38] Tamirou F Lauwerys BR Dall'Era M Mackay M Rovin B Cervera R. A proteinuria cut-off level of 0.7 g/day after 12 months of treatment best predicts long-term renal outcome in lupus nephritis: data from the MAINTAIN Nephritis Trial. Lupus Sci Med. (2015) 2:e000123. 10.1136/lupus-2015-000123 26629352 PMC4654096

[39] Dall'Era M Cisternas MG Smilek DE Straub L Houssiau FA Cervera R. Predictors of long-term renal outcome in lupus nephritis trials: lessons learned from the Euro-Lupus nephritis cohort. Arthritis Rheumatol. (2015) 67:1305–13. 10.1002/art.39026 25605554

[40] Korbet SM Lewis EJ Collaborative Study Group. Severe lupus nephritis: the predictive value of a ≥ 50% reduction in proteinuria at 6 months. Nephrol Dial Transplant. (2013) 28:2313–8. 10.1093/ndt/gft201 23787551

[41] Ugolini-Lopes MR Seguro LPC Castro MXF Daffre D Lopes AC Borba EF. Early proteinuria response: a valid real-life situation predictor of long-term lupus renal outcome in an ethnically diverse group with severe biopsy-proven nephritis? Lupus Sci Med. (2017) 4:e000213. 10.1136/lupus-2017-000213 29238603 PMC5724342

[42] Moroni G Gatto M Tamborini F Quaglini S Radice F Saccon F. Lack of EULAR/ERA-EDTA response at 1 year predicts poor long-term renal outcome in patients with lupus nephritis. Ann Rheum Dis. (2020) 79:1077–83. 10.1136/annrheumdis-2020-216965 32503858

